# Supplementary material for: Restoring brain connectivity by phrenic nerve stimulation in sedated and mechanically ventilated patients
Source: Commun Med (Lond). 2024 Nov 18;4:235. doi: 10.1038/s43856-024-00662-0 (PMC11574298; doi:10.1038/s43856-024-00662-0)

Supplementary Information

Supplementary Table 1. Main patient characteristics.

Patient Number	Age*	Sex	Disease for ICU admission	Weight (kg)	Ideal Weight (kg)	Mean Arterial Pressure (mmHg)	Norepinephrine (μg/kg/min)	Sufentanil (μg/h)	Propofol (mg/h)	Midazolam (mg/h)	Duration of Phrenic Nerve Stimulation (min)	Pa_2O_2/FiO_2 (enrolment)	Pa_2O_2/FiO_2 (normal)	Tidal Volume (mL)	Tidal Volume (mL/kg of predicted body weight)	FiO_2 (%)	Richmond Agitation Sedation Scale	Notes
1	80-86	M	Aspiration	45	57	90	0.44	15	200	0	120	185	>400	350	6.2	40	-5	cardiac arrest and acute hypoxic-ischemic encephalopathy
2	60-66	M	COVID-19	86	77	81	0.08	15	250	15	120	158	>400	400	5.2	60	-5	-
3	48-54	F	Pancreatitis	110	63	95	0.00	20	100	5	120	116	>400	375	5.9	40	-5	codeine addiction and alcoholism
4	54-60	M	Multi-Resistant Staphylococcus Aureus	65	75	75	0.36	10	300	0	120	187	>400	430	5.7	50	-5	epilepsy
5	48-54	M	Broncho-Aspiration	85	75	90	0.00	15	300	30	120	159	>400	450	6.0	50	-4	neuropath, morphine and benzodiazepine addiction
6	44-50	M	Pneumonia	128	84	75	0.04	15	250	0	120	163	>400	450	5.3	50	-4	-

***range in years to provide patients additional anonymization.**

Supplementary Table 2. Medians of heart rate and mean arterial pressure during the sessions.

Physiological Parameters (median, IQR)	Session 1	Session 2	Session 3	Session 4	p-value
Heart Rate, min^{-1}	72 (58-91)	77 (63-91)	77 (60-90)	76 (63-89)	0.9397
Mean Arterial Pressure, mmHg	83 (77-96)	85 (78-96)	83 (78-100)	83 (77-97)	0.8791

Supplementary Figure 1. Heatmaps of the p-values between the paced vs unpaced sessions for delta power frequency showing a connection between the insula (I) and lateral prefrontal cortex (LPC) during paced sessions and between posterior cingulate gyrus (PCG) and diaphragm somatosensory cortex (DSC).

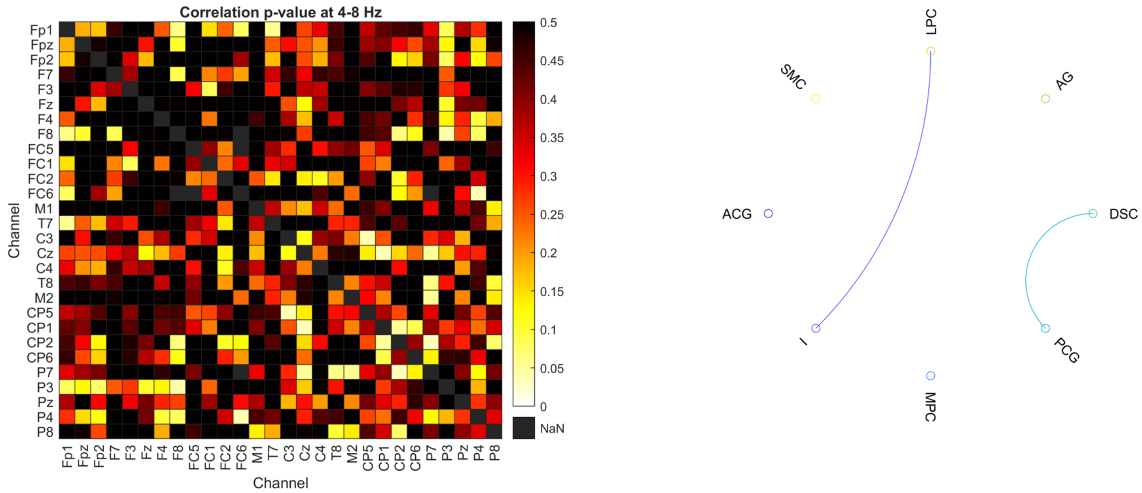

Supplement: Supplementary file 2 — Supplementary Information [file 43856_2024_662_MOESM2_ESM.pdf]
