# Supplementary material for: Restoring brain connectivity by phrenic nerve stimulation in sedated and mechanically ventilated patients
Source: Commun Med (Lond). 2024 Nov 18;4:235. doi: 10.1038/s43856-024-00662-0 (PMC11574298; doi:10.1038/s43856-024-00662-0)
Supplement: Supplementary file 3 — Description of Additional Supplementary Files [file 43856_2024_662_MOESM3_ESM.pdf]

Description of additional supplementary files

File name: Supplementary Data 1

File description: The source data for Figure 1

File name: Supplementary Data 2

File description: The source data for Figure 2

File name: Supplementary Data 3

File description: The source data for Figure 3

File name: Supplementary Data 4

File description: The source data for Figure 4
